# Musical Hallucinations Treated with Acetylcholinesterase Inhibitors

**DOI:** 10.3389/fpsyt.2015.00046

**Published:** 2015-04-07

**Authors:** Jan Dirk Blom, Jan Adriaan F. Coebergh, René Lauw, Iris E. C. Sommer

**Affiliations:** ^1^Parnassia Psychiatric Institute, The Hague, Netherlands; ^2^Department of Psychiatry, University of Groningen, Groningen, Netherlands; ^3^Department of Neurology, Ashford and St. Peter’s Hospitals, Chertsey, UK; ^4^Department of Neurology, St. George’s Hospital, London, UK; ^5^Department of Psychiatry, University Medical Center Utrecht, Utrecht, Netherlands; ^6^Rudolf Magnus Institute of Neuroscience, Utrecht, Netherlands

**Keywords:** auditory Charles Bonnet syndrome, cholinergic system, deafferentiation, donepezil, hearing loss, Oliver Sacks’ syndrome, release hallucination, rivastigmine

## Abstract

Musical hallucinations are relatively rare auditory percepts which, due to their intrusive nature and the accompanying fear of impending mental decline, tend to cause significant distress and impairment. Although their etiology and pathophysiology appear to be heterogeneous and no evidence-based treatment methods are available, case reports indicate that acetylcholinesterase inhibitors may yield positive results in patients with comorbid hearing loss. We present two female patients (aged 76 and 78 years) both of whom suffered from hearing impairment and practically incessant musical hallucinations. Both patients were successfully treated with the acetylcholinesterase inhibitor rivastigmine. Based on these two case descriptions and an overview of studies describing the use of acetylcholinesterase inhibitors in similar patients, we discuss possible mechanisms and propose further research on the use of acetylcholinesterase inhibitors for musical hallucinations experienced in concordance with hearing loss.

## Introduction

Musical hallucinations (also known as musical hallucinosis, auditory Charles Bonnet syndrome, and Oliver Sacks’ syndrome) are characterized by hallucinated songs, tunes, melodies, harmonics, rhythms, and/or timbres (1). They can be perceived within the head, or as though emanating from the environment. However, by definition, they are perceptual in nature and thus different from the “earworms” or “tunes in the head” that we all experience at times (2). When first perceiving musical hallucinations, people tend to attribute them to an external source but, within a few days, most realize that the music originates from “within” their head. Insight is often intact and, apart from hearing loss or tinnitus, most patients display no additional comorbidity. Therefore, the term “idiopathic musical hallucination” is used to describe such cases, in contrast to those which are attributed to demonstrable underlying pathology, i.e., symptomatic musical hallucinations.

The prevalence of musical hallucinations seems to be higher than traditionally suspected, even when taking into account a review by Cope and Baguley (3) who reported their presence in almost 1% of individuals in a population with acquired hearing loss. Experienced clinicians report relatively frequent encounters with people experiencing them (4), and a survey among patients referred for audiometric testing found musical hallucinations in 3.6% of the cases (5). The pathophysiology of such hallucinations is probably diverse and certainly needs further elucidation. A magnetoencephalography (MEG) study in one individual with musical hallucinations and hearing loss indicates involvement of right temporoparietal areas (6), whereas a more recent MEG study in a similar patient indicates involvement of the left anterior superior temporal gyrus, motor cortex, posteromedial cortex, and left lateral orbitofrontal cortex at the onset of hallucinations after a residual inhibition paradigm (7). However, apart from those specific areas, the vast brain network involved in their mediation seems to comprise auditory areas, basal ganglia, brainstem, pons, tegmentum, cerebellum, hippocampi, amygdala, visual areas and, in some cases, perhaps also the peripheral auditory system (4). The risk factors for musical hallucinations are also complex and diverse (Table 1).

**Table 1:** **Known risk factors for musical hallucinations: after Sacks and Blom (4)**.

Hearing impairment
Tinnitus
Older age
Female sex (possibly)
Cerebral pathology
Epilepsy
Brain tumor
Stroke
Hemorrhage
Meningitis
Neurodegenerative disease (Alzheimer’s disease, Lewy body dementia)
Neurosyphilis
Localized atrophy
Traumatic lesion
Psychiatric disorder
Schizophrenia spectrum disorder
Bipolar disorder
Psychotic depression
Depression
Obsessive–compulsive disorder
Adaptation impairment
Personality disorder
ADHD
Cocaine dependence
Intoxication
Alcohol
Antidepressants
Opioids
Antibiotics
Beta blockers
Quinine
Salicylates
Miscellaneous
Behçet’s disease
Hashimoto’s encephalopathy
Lyme disease
Electroconvulsive treatment
Cochlear implantation
Sensory deprivation

The main risk factors for musical hallucinations are impaired hearing, tinnitus, advanced age and, perhaps, also female sex; however, the latter finding may be due to an overrepresentation of females in the literature (4). It remains uncertain whether psychosis, schizotypal or schizoid personality, and other psychiatric disorders increase the risk for musical hallucinations (8–11).

Evidence-based treatment protocols are lacking. However, case reports and small case series indicate that some people can be treated non-pharmacologically through psychoeducation, use of a hearing aid, and/or attention-diverting activities, whereas others can be treated pharmacologically with anticonvulsants, antidepressants, or antipsychotics; however, in many cases, the hallucinations prove refractory to treatment (4, 12). Here, we present two patients who derived benefit from the acetylcholinesterase inhibitor rivastigmine. Based on these two cases and a discussion of similar earlier cases, we explore possible mechanisms of action for acetylcholinesterase inhibitors in the treatment of musical hallucinations.

## Materials and Methods

We describe two patients, the first of whom is a 76-year-old female who was treated at the outpatient clinic of Parnassia Psychiatric Institute (The Hague, the Netherlands). As this patient died at age 80 years, written consent to publish was obtained from her son. The second patient is a 78-year-old female who was treated at Ashford/St. Peter’s Hospital (Chertsey, UK). Due to her sudden death no consent to publish could be obtained. For the present review, we conducted a systematic search in Pubmed and the Ovid database, which included EMBASE (1980 through November 2014), Ovid Medline (1948 through November 2014), and PsycINFO (1806 through November 2014). In each database, the search terms musical hallucination, musical hallucinosis, and auditory Charles Bonnet syndrome, were used separately. Each of the terms was then combined separately with cholinesterase inhibitor, acetylcholinesterase inhibitor, rivastigmine, and donepezil.

## Results

### Case reports

#### Patient 1

In 2009, a 76-year-old woman with impaired hearing was referred because of musical hallucinations, which she had experienced since her husband’s death 6 years earlier. On the day of his death, she had suddenly heard hymns, lullabies, pop songs, and classical tunes, which repeated themselves indefinitely before changing into different pieces of music. Although she perceived them inside the head, they had a definite perceptual quality. They were present from the moment she awoke until late at night, and she used a radio to drown them out when trying to sleep. During the daytime, they receded into the background only when she carried out tasks that required her full attention. Because she and her husband had shared a fondness for the same music, she initially considered the hallucinations to be a sign of ongoing interconnectedness between the two of them. This conviction was strengthened by various hypnagogic episodes during which she once saw her husband surrounded by a bright light, and on other occasions felt a presence nearby or a hand through her hair. After 6 months, the musical hallucinations had exhausted her to such an extent that she consulted a psychologist, who interpreted them as part of a normal grieving process and offered her counseling – which she quit after a few sessions. When she consulted our group 5 years later, she was not receiving any psychiatric treatment. Her somatic history comprised tension headaches, asthma, arthritis, and a myocardial infarction. Her medication consisted of acetylsalicylate, dipyridamole, pantoprazole, isosorbide mononitrate, simvastatin, salbutamol, and tiotropium bromide. We found no evidence for any of these medications to be a potential cause of her hallucinations, except for the acetylsalicylate (13). The psychiatric examination yielded no abnormalities other than the grief-related hypnagogic phenomena described above. Notably, there were no signs of other hallucinations, depression, psychosis, schizotypy, or cognitive decline (although the latter was not formally tested). The neurological examination and the electroencephalogram (EEG) were also unremarkable. Magnetic resonance imaging (MRI) of the brain showed multiple white-matter lesions, especially in the right inferior frontal area and the left parietal lobe, but nothing unusual for her age. To rule out the possibility that the hallucinations were caused by the use of acetylsalicylate, the latter drug was changed to clopidogrel, but without any effect. Despite the negative EEG findings, we then prescribed 150 mg of valproic acid; with this treatment, the musical hallucinations diminished substantially and our patient experienced a range of symptom-free hours every day. We also advised her to use her hearing aid, to optimize the use of her radio, and to engage in social events; however, these latter activities had no effect. Meanwhile, because a higher dose of valproic acid was not well tolerated, in an attempt to improve her condition we tapered it off and proposed rivastigmine instead. As in dementia, we titrated the rivastigmine dose upwards; however, because our patient reported severe dizziness at a dosage of 4.5 mg, we advised her to reduce the dose to 1.5 mg/day. Within a few days, the musical hallucinations had vanished completely. At this low dose, there were no side-effects and, during a follow-up period of 4 years until her death in 2013 due to a cerebral hemorrhage, she remained symptom-free, even though she occasionally missed a dose of rivastigmine.

#### Patient 2

In 2014, a 78-year-old woman with hearing impairment was referred because of musical hallucinations, which had been present for 1 year. Initially, she was convinced that the people next door kept playing Christmas carols, even at night and when they had left the house. Later, the experienced music was unrecognizable to her. She felt she wanted to harm the neighbors, and also became estranged from her sister who maintained that she did not hear the music. Despite also hearing it when she stayed at her daughter’s home, she continued to believe that the neighbors were causing the noise. Adjusting the hearing aids that she had worn for 1 year brought no relief, but watching TV reduced the volume of the hallucinated music. She scored the usual level of loudness on a visual analog scale (VAS) as 8–9/10 and stated that the noise interfered with her falling asleep. Our patient had lived alone for 20 years and on most days did not speak to anyone. Although there was no psychiatric history, due to the hallucinations she developed a depressed mood, which did not improve significantly with mirtazapine. Her other medications comprised metformin, gliclazide, sitagliptin, co-codamol, simvastatin, and atenolol, neither of which could be held responsible for her hallucinations. There were no abnormalities on neurological and psychiatric examination. Notably, we found no evidence for any other types of hallucination. In the consulting room, the musical hallucinations commenced after 30 s of silence. The Addenbrooke’s Cognitive Examination-Revised (ACE-R) score was 79/100, suggesting mild cognitive impairment (MCI). An MRI scan of the brain showed unremarkable white-matter changes. Within days of starting rivastigmine 1.5 mg, the music became less frequent and less loud (VAS 5/10) and her anger and depressed mood disappeared. We did not repeat ACE-R, but insight into the origin of the music improved significantly even though at times she still suspected the neighbors. Three months after starting the rivastigmine, in the consulting room the music started after 1 min, rather than after 30 s, of silence. The dose was increased to 4.5 mg/day in an attempt to treat both the hallucinations and MCI, but 6 months later she died suddenly due to pneumonia.

### Literature search

Our search yielded 210 papers on musical hallucinations and 72,999 papers on acetylcholinesterase inhibitors. Of those papers, three described four original cases involving the successful treatment of musical hallucinations with the acetylcholinesterase inhibitor donepezil (14–16). A fourth paper (6), which apparently described one of those four cases (14) at an earlier stage, was omitted. Table 2 provides a summary of the four original cases plus a summary of our two present cases.

**Table 2:** **Overview of reported cases of musical hallucinations treated with acetylcholinesterase inhibitors**.

Reference	Patient	Content of MHs	Tinnitus	Hearing loss	Comorbid disorders	Results of auxiliary investigations	Treatment	Clinical efficacy	Duration of follow-up
Ukai et al. (14)	F, 82 years	Old familiar songs accompanied by musical instruments which later evolved into monotonous, simple melodies	−	+	−	MRI: diffuse mild cortical atrophy consistent with aging	Donepezil 5 mg/day (plus polypharmacy)	Almost complete remission	5 years
Strauss and Gertz (15)	M, 54 years	Songs sung by a male voice, sometimes accompanied by piano or guitar, and sometimes orchestral music	+	+	Hemiparesis due to infarctions of the bilateral medial cerebral artery territories	MRI: extensive ischemic lesions in the right medial cerebral artery territory, including insular cortex and basal ganglia, and left occipital ischemic lesions	Donepezil 5 mg/day	Complete remission	8 weeks
Strauss and Gertz (15)	M, 78 years	Christmas carols and folk songs, sung by a male voice	+	+	−	MRI: symmetric diffuse brain atrophy and periventricular microangiopathic changes	Donepezil 5 mg/day	Complete remission	4 months
Zilles et al. (16)	F, 90 years	Choirs and brass music	−	+	Reactive depression	MRI: global brain atrophy, bifrontal hygromas, and a leukodystrophia-like brain parenchyma	Donepezil 5 mg/day	Complete remission	1 week
Blom et al. (17)	F, 76 years	Hymns, lullabies, pop songs, and classical tunes	−	+	Myocardial infarction	MRI: white-matter lesions, especially in the right inferior frontal area and the left parietal lobe	Rivastigmine 1.5 mg/day (plus polypharmacy)	Complete remission	4 years
Blom et al. (17)	F, 78 years	Christmas carols and (later) unrecognizable music	−	+	−	MRI: white-matter lesions, non-specific	Rivastigmine 1.5 mg/day (plus polypharmacy)	Partial remission	3 months

*MHs, musical hallucinations; F, female; M, male*.

## Discussion

All six cases published so far are consistent with the definition of idiopathic musical hallucinations, even though one of the patients described by Strauss and Gertz (15) experienced an intensification of preexisting musical hallucinations after a stroke. Regarding their pathophysiology, such hallucinations are conceptualized as deafferentiation phenomena, i.e., release hallucinations evoked by the relative absence of normal auditory input (4, 14). As noted by Ukai et al. (14), age-dependent dysfunction of cholinergic neurons may be a predisposing condition for the mediation of such release phenomena.

The brain’s cholinergic system is mainly involved not only in attention, learning, and memory but also in visual perception and visual attention (the latter being modulated by gamma oscillations and possibly also by low-frequency alpha/beta oscillations under the influence of ACh in visual cortex) (18). Thus, there is substantial evidence for the efficacy of acetylcholinesterase inhibitors in the treatment of visual hallucinations experienced in the context of Parkinson’s disease (19–21). In addition, there is evidence for their efficacy in instances of isolated visual hallucinations. For example, Ukai et al. (22) described a patient with isolated visual hallucinations in the absence of cognitive decline who was successfully treated with donepezil; also, our group reported the successful treatment of visual disorders of perception with rivastigmine (17).

Animal studies suggest that cortical cholinergic input is also required for normal auditory perception (23). In humans, Irimajiri et al. (24) found that acetylcholinesterase inhibitors alter auditory cortical somatosensory potentials (N20, P50) in treated versus untreated MCI patients, while a study among Alzheimer patients indicates that they also alter resting-state EEG rhythms (25). Our knowledge of ACh in the perception, storage, production, and reproduction of music is still in its infancy, even though its involvement in auditory learning and memory has been established in animal studies and implications for neuromusic research are considered imminent (26). The role of ACh in the processing of music is supported by case reports on potent anticholinergic antidepressants (such as amitriptyline) and their ability to evoke musical hallucinations (9, 27), and on studies of musical hallucinations in association with degenerative brain diseases characterized by cholinergic deficits, such as Alzheimer’s disease and Lewy body disease (19, 28).

The receptor sites responsible for the mechanism of action of acetylcholinesterase inhibitors in cases of musical hallucination are as yet unknown. Postsynaptically, ACh acts upon nicotinic and muscarinic acetylcholine receptors. Acetylcholinesterase inhibitors increase the presynaptic availability, as well as the duration of action of ACh, by preventing the acetylcholinesterase enzyme from breaking this neuromodulator down. Rivastigmine, a brain-selective “pseudo-irreversible” acetylcholinesterase inhibitor, prevents the breakdown of ACh for a duration of 10 h (29). Animal studies have shown that rivastigmine has a specific effect on cortex and hippocampus (30). Because musical hallucinations often present in the form of familiar songs and other pieces remembered from the past (31), the hippocampus with its central role in memory and emotion seems a likely candidate. That hypothesis is further supported by the deactivation of the hippocampus in functional MRI studies of verbal auditory hallucinations (32). In addition, posterior medial cortex (with its role in memory and perception) may be involved in the retrieval of musical melody from memory (7). Other candidate mechanisms include those found by Kumar et al. (7), i.e., an increase in the gamma band in left anterior superior temporal gyrus with stronger hallucinations (which is normally involved in melody perception), in the beta band in motor cortex and posteromedial cortex, and in the theta/alpha bands in left lateral orbitofrontal cortex (associated with unpleasant music).

### Conclusion and limitations

Although our knowledge of ACh and its role in the processing of music needs further elucidation, there are reasons to consider acetylcholinesterase inhibitors for pharmacoclinical research in musical hallucinations associated with hearing loss. First of all, they seem to be safer and to yield fewer side-effects than antipsychotics, antidepressants, and anticonvulsants (33). Second, they may even yield beneficial side-effects when administered to patients suffering from comorbid cognitive decline. Furthermore, a beneficial effect may be obtained relatively quickly. For example, in the six patients described above, acetylcholinesterase inhibitors were effective within a few days (i.e., much faster than the antipsychotics used in patients experiencing verbal auditory hallucinations), at a low dose (rivastigmine 1.5 mg is much lower than the 9 mg dose used for dementia), and with substantial effect. Nevertheless, most of the evidence for the involvement of ACh in the mediation of musical hallucinations is indirect and based on observations among very small numbers of patients. Moreover, there may be some publication bias as musical hallucinations refractory to treatment with acetylcholinesterase inhibitors may have remained unreported. For other types of musical hallucination, especially those not associated with hearing loss and/or deafferentiation, the efficacy of these drugs has not yet been established.

## Author Contributions

JB designed the manuscript, acquired data for the first case description, drafted the manuscript, gave final approval of the version to be published, and agreed to be accountable for all aspects of the work in ensuring that questions related to the accuracy or integrity of any part of the work are appropriately investigated and resolved. JC contributed substantially to the design of the manuscript, acquired data for the second case description, revised the manuscript, gave final approval of the version to be published, and agreed to be accountable for all aspects of the work in ensuring that questions related to the accuracy or integrity of any part of the work are appropriately investigated and resolved. RL acquired data for the first case description, revised the manuscript, gave final approval of the version to be published, and agreed to be accountable for all aspects of the work in ensuring that questions related to the accuracy or integrity of any part of the work are appropriately investigated and resolved. IS contributed substantially to the design of the manuscript, revised the manuscript, gave final approval of the version to be published, and agreed to be accountable for all aspects of the work in ensuring that questions related to the accuracy or integrity of any part of the work are appropriately investigated and resolved.

## Conflict of Interest Statement

The research was conducted in the absence of any commercial or financial relationships that could be construed as a potential conflict of interest.
